# Endogenous Opioid Signaling in the Mouse Retina Modulates Pupillary Light Reflex

**DOI:** 10.3390/ijms22020554

**Published:** 2021-01-08

**Authors:** Allison M. Cleymaet, Casey-Tyler Berezin, Jozsef Vigh

**Affiliations:** 1 Department of Biomedical Sciences, Colorado State University, Ft. Collins, CO 80523, USA; allison.cleymaet@colostate.edu; 2 Department of Clinical Sciences, Colorado State University, Ft. Collins, CO 80523, USA; 3 Cellular and Molecular Biology Graduate Program, Colorado State University, Ft. Collins, CO 80523, USA; CT.Berezin@colostate.edu

**Keywords:** opioids, retina, melanopsin, intrinsically photosensitive ganglion cell, pupillary light reflex

## Abstract

Opioid peptides and their receptors are expressed in the mammalian retina; however, little is known about how they might affect visual processing. The melanopsin-expressing intrinsically photosensitive retinal ganglion cells (ipRGCs), which mediate important non-image-forming visual processes such as the pupillary light reflex (PLR), express β-endorphin-preferring, µ-opioid receptors (MORs). The objective of the present study was to elucidate if opioids, endogenous or exogenous, modulate pupillary light reflex (PLR) via MORs expressed by ipRGCs. MOR-selective agonist [D-Ala^2^, MePhe^4^, Gly-ol^5^]-enkephalin (DAMGO) or antagonist D-Phe-Cys-Tyr-D-Trp-Arg-Thr-Pen-Thr-NH_2_ (CTAP) was administered via intravitreal injection. PLR was recorded in response to light stimuli of various intensities. DAMGO eliminated PLR evoked by light with intensities below melanopsin activation threshold but not that evoked by bright blue irradiance that activated melanopsin signaling, although in the latter case, DAMGO markedly slowed pupil constriction. CTAP or genetic ablation of MORs in ipRGCs slightly enhanced dim-light-evoked PLR but not that evoked by a bright blue stimulus. Our results suggest that endogenous opioid signaling in the retina contributes to the regulation of PLR. The slowing of bright light-evoked PLR by DAMGO is consistent with the observation that systemically applied opioids accumulate in the vitreous and that patients receiving chronic opioid treatment have slow PLR.

## 1. Introduction

Over the past 25 years, the liberalization of laws governing opioid prescription for the treatment of chronic non-cancer pain has led to dramatic increases in opioid use, often referred to as an opioid epidemic in the United States [1,2,3]. While there exist several biomarkers for opioid effect, in humans, the development of pupil constriction present under constant light conditions (“resting miosis”) is used as an indicator of systemic opioid effect [4,5,6,7,8,9]. However, the effect of opioids on resting pupil diameter is highly variable and species-dependent, in some species causing dilatation under constant light conditions (“resting mydriasis”) and constriction in others [7]. Opioids also exert species-specific though less variable effects on the pupillary light reflex (PLR), e.g., retarding the PLR in most species, including humans [8,9] and cats [10,11] and yet enhancing it in the rabbit [12]. It is noteworthy that the PLR evoked by bright blue light in chronic opioid user patients has a reduced velocity [4]. Given that tolerance of the PLR to a light flash develops at a different rate than that of pupil diameter [13], it is generally held that different mechanisms control resting pupil size and the magnitude of the PLR [8,11,13,14,15]. These mechanisms diverge at the level of the midbrain. Resting pupil diameter is controlled by tonic firing of the Edinger-Westphal nucleus(EWN) (or the oculomotor nucleus (OMN)), which is spontaneous and persistent in the face of deafferentation. The PLR, however, is determined by retinal illumination, and subsequent light evoked EWN excitation and an increase in the firing rate of parasympathetic neurons arising from the EWN and innervating the iris via the short ciliary nerve [14].

The afferent arm of the PLR is mediated by a subset of inner retinal ganglion cells, namely the intrinsically photosensitive retinal ganglion cells (ipRGCs) that contain the photopigment melanopsin, which is most sensitive to intense, short-wavelength (blue) light [16,17,18]. The axons of ipRGCs that also express the nuclear factor Brn3b innervate the olivary pretectal nucleus (OPN), which mediates the PLR via the EWN [19,20,21,22,23,24]. Both classical photoreceptors, rods and cones, and the ipRGCs contribute to the PLR. Rodless, coneless mice maintain normal PLRs in response to high irradiance stimuli. [16,25]. Melanopsin knockout (KO) mice maintain normal PLRs in response to low irradiance stimuli but not high, with melanopsin being requisite for maximal constriction, [17,25]. In addition, treatment with opsinamides, antagonists of melanopsin-mediated phototransduction, slowed pupil constriction starting 1 **s** after onset of high irradiance stimuli [26]. Triple KO mice, i.e., mice lacking both classical photoreceptor transduction mechanisms and melanopsin, do not manifest a PLR, indicating the complementary nature of both systems [25,27]. However, outer retinal signals contribute to the PLR via the conduit of ipRGCs, as genetic ablation of ipRGCs eliminates rod–cone-mediated pupil constriction in response to all light intensities [28]. It is of note that the photoresponses of rods, cones, and ipRGCs are not linearly additive, as the melanopsin photoresponse exclusively drives the PLR given stimuli above the threshold of the melanopsin photoresponse (480 nm, 10^11.5^ photons/cm^2^/s) [17], effectively shunting rod–cone-mediated outer retinal signals that feed into the ipRGCs. Below this threshold, after a brief period of adaptation, tonic rod signaling synergistically drives the PLR via downstream, central ipRGC glutamatergic output, maintaining pupil constriction at irradiances below the melanopsin threshold and enhancing sensitivity to long-wavelength light [29,30]. In contrast, cones minimally contribute to maintaining pupil constriction at either high or low irradiances [29], unless they are permitted to dark adapt with short, intermittent dark pulses [31].

Our prior work demonstrated that in the adult mouse retina, the opioid peptide β-endorphin is expressed by cholinergic amacrine cells [32] and that opioids, via β-endorphin-preferring opioid receptors (MORs), strongly attenuate the light-evoked firing of ipRGCs in mice and rats [33]. Modulatory processes that are capable of inhibiting ipRGC activity have been proposed to inhibit ipRGC-mediated, light-driven behavior [26]. In the present study, we test the hypothesis that inhibition of light-evoked ipRGC signaling via MORs modulates the murine PLR. We also determine the relative impact of opioids on classical photoreceptor vs. ipRGC contributes to the PLR by means of focal, intraocular applications of MOR selective agonist and antagonist in combination with transgenic mouse lines lacking MORs exclusively in ipRGCs or systemically. Our data suggest that endogenous opioids modulate the PLR by reducing its magnitude in response to dim light in the dark-adapted retina, whereas exogenous opioids eliminate dim-light-evoked PLR. Furthermore, intraocular applications of MOR-selective agonist DAMGO slowed the bright-blue-light-evoked PLR. Although MOR expression by ipRGCs in humans has not been confirmed, this latter finding appears to be consistent with the observations that opioids accumulate in the vitreous of the human eye upon systemic delivery [34,35], and patients with chronic opioid treatment history have slow bright-blue-light-evoked PLR [4].

## 2. Results

### 2.1. MOR-Specific Agonist DAMGO Inhibited Dark-Adapted Pupillary Light Reflex (PLR) in Wild-Type (WT) Mice

**Figure 1:**
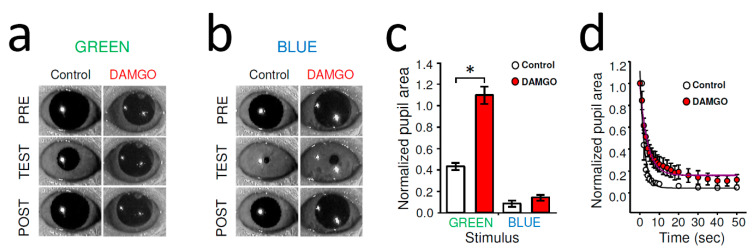
DAMGO eliminated rod/cone-driven PLR and slowed melanopsin-dependent PLR in WT mouse. (**a**) Representative picture of stationary mouse PLR (TEST) in control evoked by rod/cone activation (10^10^ photons/cm^2^/s at 525 nm, GREEN). Note the wide-open dark-adapted pupil just before and 2 min after the light stimulation (PRE and POST, respectively). Intraocular injection of DAMGO (2 μL of 2 mg/mL) eliminated PLR. (**b**) Bright blue photopic irradiance that activated melanopsin signaling (10^14^ photons/cm^2^/s at 470 nm, BLUE) evoked strong pupil constriction in control (TEST) that was minimally affected by intraocular delivery of DAMGO. (**c**) Quantification of stationary PLR data. AVG ± SEM; control: *n* = 16; DAMGO: *n* = 9; * *p* < 0.001, Student’s *t*-test. (**d**) Detailed analysis of the pupil constriction triggered by blue light revealed that although the overall pupil constriction was not reduced, DAMGO markedly slowed the PLR. Data are shown as AVG ± SEM and fit by exponential decay functions.

Dark-adapted PLR mediated by classical photoreceptors was evoked by stimulating the right eye with green light (λ = 525 nm) at an intensity (10^11^ photons/cm^2^/s) that saturates rods and activates cones [36] but remains below the melanopsin activation threshold of ~10^13.5^ photons/cm^2^/s at 525 nm. [16,17,18]. The second stimulus (10^14^ photons/cm^2^/s at 470 nm) was well above melanopsin threshold (10^11.5^/photons/cm^2^/s at 480 nm) [17,18], to activate ipRGCs directly. In dark-adapted WT mice, unilateral, intraocular injection of DAMGO (2 µL of 2 mg/mL) strongly inhibited contralateral rod/cone-driven PLR; in fact, after the DAMGO administration, the light stimulation did not trigger any noticeable constriction (Figure 1a). The normalized pupil area of green-light-evoked stationary PLR was significantly greater after DAMGO injection compared to control (control: 41.78 ± 3.16%, *n* = 16, DAMGO: 107.77 ± 5.56%, *n* = 9, *p* < 0.001, Student’s *t*-test) (Figure 1c). The stationary PLR evoked by bright blue irradiance that can activate melanopsin signaling directly was only slightly inhibited by DAMGO (Figure 1b), but this effect was not found to be statistically significant (8.67 ± 3.02%, *n* = 5, DAMGO: 14.18 ± 2.67%, *n* = 5, *p* = 0.82, Student’s *t*-test) (Figure 1c). However, a more detailed analysis addressing the dynamics of the bright blue light evoked PLR showed a marked slowing of the pupil constrictions under DAMGO conditions compared to control (Figure 1d).

### 2.2. Dark-Adapted MOR-Deficient Mice Showed Normal PLR, but Intraocular DAMGO Had No Effect on Their PLR

To elucidate whether the intraocularly administered MOR-selective agonist DAMGO effect on the pupillary light reflex was mediated by MORs expressed by ipRGCs, we performed experiments on mice lacking MORs entirely (MKO) as well as on mice lacking MORs only in ipRGCs (McKO).

**Figure 2:**
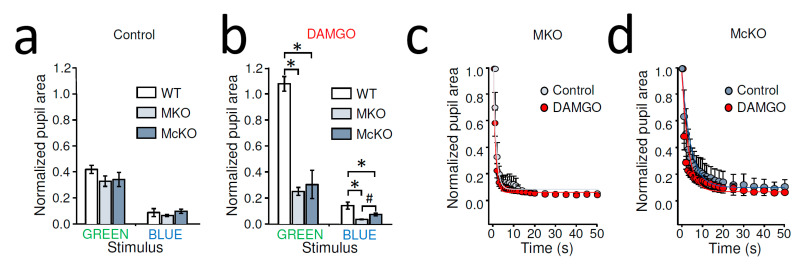
Effects of DAMGO on rod/cone driven and melanopsin-dependent PLR in MKO and McKO mouse: (**a**) Control stationary PLR of dark-adapted MKO and McKO mice was not significantly different from that of WT mice in response to a light stimulus activating rods/cones (10^10^ photons/cm^2^/s at 525 nm, GREEN) or melanopsin signaling (10^14^ photons/cm^2^/s at 470 nm, BLUE). AVG ± SEM. (**b**) PLRs of dark-adapted MKO and McKO mice that received a unilateral, intraocular injection of DAMGO were significantly different from those of WT mice receiving the same treatment. Furthermore, under these conditions, blue light evoked stronger PLR in MKO vs. McKO mice. * *p* < 0.001, # *p* = 0.02, one-way ANOVA with All Pairwise Multiple Comparison, Holm-Sidak method. (**c**) No difference was found between control and DAMGO treatment in the blue-light-evoked PLR kinetics of MKO mice. Data are shown as AVG ± SEM (*n* = 10) and fit by exponential decay functions. (**d**) Detailed analysis of the pupil constriction triggered by blue light revealed no difference in PLR of McKO mice due to DAMGO treatment. AVG ± SEM (control: *n* = 9, DAMGO: *n* = 11) and fit by exponential decay functions.

Control stationary PLR of dark-adapted MKO and McKO mice was not significantly different from that of WT mice for any light stimulus (normalized pupil area of green light WT: 41.78 ± 3.16%, *n* = 16, MKO: 32.74 ± 3.98%, *n* = 21, McKO: 34.08 ± 5.38, *n* = 9, *p* = 0.55, one way ANOVA; blue light WT: 8.67 ± 3.02%, *n* = 5, MKO: 6.47 ± 0.80%, *n* = 15, McKO: 9.74 ± 1.60%, *n* = 9, *p* = 0.22, one way ANOVA) (Figure 2a). MKO and McKO mice are thus valid models for the assessment of acute MOR-mediated inhibition of ipRGCs on PLRs.

As opposed to dark-adapted WT mice, unilateral, intraocular injection of DAMGO (2 µL of 2 mg/mL) did not inhibit contralateral green-light-evoked PLR in MKO or McKO mice; in other words, in the presence of DAMGO, our green light stimulus triggered PLR in MKO or McKO mice so that the pupil sizes of the transgenic mice were significantly different from those measured in WT following intraocular DAMGO injection (*p* < 0.001), whereas the PLR in MKO and McKO animals was similar (*p* = 0.534, one way ANOVA with All Pairwise Multiple Comparison, Holm-Sidak method)(Figure 2b). Similarly, following intraocular DAMGO, the bright blue light caused more complete PLR in both MKO and McKO mice than what was observed in WT (WT: 14.17 ± 2.67%, *n* = 5; MKO: 3.52 ± 0.30%, *n* = 10; McKO: 7.26 ± 1.06%, *n* = 11, *p* < 0.001, one way ANOVA). Furthermore, we found the blue light evoked PLR stronger in MKO mice compared to that in McKOs (*p* = 0.02, All Pairwise Multiple Comparison, Holm-Sidak method) (Figure 2b).

Importantly, when compared to the normalized pupil area measures in control and DAMGO-treated MKO animals, we found no significant statistical difference for either stimuli (green light control constriction: 32.74 ± 3.98%, *n* = 21, green light DAMGO: 24.98 ± 3.08%, *n* = 12, *p* = 0.19, Student’s *t*-test; blue light control: 5.23 ± 0.66%, *n* = 9, blue light DAMGO: 3.52 ± 0.31%, *n* = 9, *p* = 0.06, paired Student’s *t*-test). Similarly, in McKO mice, the intraocular DAMGO injection appeared to be ineffective at altering PLR (normalized pupil area of green light control: 34.08 ± 5.38%, *n* = 9, green light DAMGO: 30.38 ± 10.90%, *n* = 6, *p* = 0.94, Student’s *t*-test; blue light control: 9.74 ± 1.60%, *n* = 9, blue light DAMGO: 7.27 ± 1.06%, *n* = 11, *p* = 0.20; Student’s *t*-test). In addition, unlike WT mice, where the intraocular DAMGO slowed the PLR (Figure 1d), detailed analysis of the dynamic PLR of MKO and McKO mice did not show apparent slowing of the blue light response under DAMGO conditions compared to control (Figure 2c,d). Taken together, these results are consistent with the notion that the MOR-selective agonist DAMGO after intraocular delivery acted on MORs expressed by ipRGCs to reduce rod/cone-driven PLR.

### 2.3. MOR Selective Antagonist CTAP Increased Dark-Adapted PLR Triggered by Rod-Saturating Green Light in WT Mice

While the lack of effect of DAMGO on PLR in MKO and McKO animals is consistent with the notion that the MOR selective agonist DAMGO after intraocular delivery acted on MORs expressed by ipRGCs to reduce rod/cone-driven PLR (Figure 2b, GREEN), the increased bright blue light evoked PLR in the presence of DAMGO in the MOR and McKO-knockout mice relative to WT is somewhat perplexing (Figure 2b, BLUE), especially when considering the data collected under similar stimulation paradigm using these mouse lines without DAMGO, where no differences were detected (Figure 2a, BLUE). It is tempting to speculate that such an enhancement could be related to a side effect of the intraocular injection itself. However, while a neurogenic reflex uveitis may result in pupil constriction in the injected eye, in our experimental design, the cause of the post-injection enhancement of PLR in the contralateral eye cannot be explained by that. Furthermore, it appears that the negative modulatory effects of DAMGO on the PLR are sufficiently potent to overcome this phenomenon, if it exists, in the WT mouse, given the absence of pupil constriction in response to rod/cone-activating stimulation in the dark-adapted WT mice following DAMGO injection (Figure 1c, GRREN, Figure 2b, GREEN). Another plausible explanation of the increased PLR in MOR-knockout mice is that endogenous opioids might be responsible for a small tonic inhibition of dark-adapted PLR, which reached significance in the DAMGO injection paradigm (Figure 2b) in response to blue light but not during control PLR tests in MKO and McKO mice (Figure 2a). To study the potential contribution of endogenous opioids to the PLR more explicitly, in WT mice, we tested the effect of MOR selective antagonist CTAP on PLR. In these experiments, dark-adapted PLR was evoked by our previously used rod-saturating/cone activating green stimulation in dark-adapted retina, whereas light-adapted (photopic) PLR was triggered by our previously used bright blue stimulation that was superimposed on the rod-saturating/cone-activating green background illumination.

**Figure 3:**
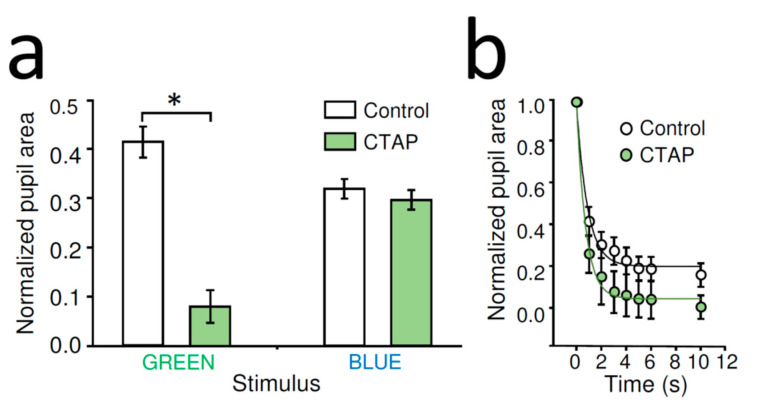
Effects of CTAP on rod/cone-driven dark adapted and melanopsin-dependent light-adapted PLR in WT mouse: (**a**) stationary contralateral PLR in response to a light stimulus activating rods/cones (10^10^ photons/cm^2^/s at 525 nm, GREEN) of dark-adapted WT mice was enhanced by intraocular CTAP administration (1 µL of 2 mg/mL). AVG ± SEM, control: *n* = 16, CTAP: *n* = 6, * *p* < 0.001 (Student’s *t*-test). The light-adapted, photopic PLR evoked by bright blue light (10^14^ photons/cm^2^/s at 470 nm, BLUE) superimposed on a rod-saturating/cone activating background illumination (10^10^ photons/cm^2^/s at 525 nm) was not altered by CTAP. AVG ± SEM, control: *n* = 10, CTAP: *n* = 5. (**b**) Detailed analysis of the rod/cone-driven pupil constriction reveal faster PLR following CTAP treatment. Data shown as AVG ± SEM, fit by exponential decay functions.

Unilateral, intraocular CTAP administration (1 µL of 2 mg/mL) significantly enhanced the contralateral rod/cone-mediated stationary PLR evoked by green light of dark-adapted WT mice compared to that of control (normalized pupil area of control: 41.78 ± 3.16%, *n* = 16, CTAP: 14.77 ± 3.32%, *n* = 6, *p* < 0.001, Student’s *t*-test) (Figure 3a). The CTAP-mediated enhancement of PLR was also associated with a slight increase in the velocity of constriction (Figure 3b). Similar unilateral, intraocular CTAP treatment did not alter significantly stationary PLR of WT mice evoked by bright blue light stimulus superimposed on rod-saturating/cone-activating background illumination (normalized pupil area of control: 37.99 ± 4.55%, *n* = 10, CTAP: 27.58 ± 2.92%, *n* = 5, *p* = 0.15, Student’s *t*-test) (Figure 3a).

## 3. Discussion

The aim of the present study was to determine the effect of modulation of ipRGC signaling via MORs on the murine PLR. The main findings of this study were as follows. (1) In WT mice but not in MKO or McKO mice, intraocular application of the MOR selective agonist DAMGO strongly inhibited rod/cone-driven PLR and slowed melanopsin-driven PLR. (2) Intraocular application of a MOR-selective antagonist CTAP enhanced rod/cone-driven PLR in the dark-adapted retina but not melanopsin-driven PLR under photopic conditions in WT mice. These results identify a novel site of action for exogenous and potentially endogenous opioids in the retina, i.e., MORs on ipRGCs, that has a significant impact on a behavioral measure of opioid effect, the PLR.

### 3.1. Pupillary Light Reflex (PLR) in Mice

The PLR consists of both sustained and transient components that are determined by the contribution of specific photoresponses. In addition to promoting maximal pupil constriction in response to high-irradiance stimuli as well as late PLR constriction velocity, melanopsin phototransduction is responsible for the post illumination pupillary response, i.e., sustained pupil constriction after light offset [37,38], as well as maintenance of pupil constriction under long-term low-irradiance photopic conditions [29]. This sustained component of the PLR, as well as stable daytime pupil diameter, is mediated by the central release of the neuropeptide pituitary adenylyl cyclase-activating polypeptide (PACAP) by ipRGCs into the brain [30]. The synaptic input generated by classical photoresponses that impinges upon the ipRGCs extends the dynamic range of the PLR in both the temporal frequency and intensity domains. Blockade of rod–cone signaling increases PLR response latency by ~1 s [37], and the pupils of patients with outer retinal blindness cannot track high-frequency intermittent light [31]. In mice without classical photoreceptor input to ipRGCs, the PLR is ~4 log units less sensitive than wild type (WT) [16,17].

Our results show that activation of MORs expressed by ipRGCs is a negative modulator of the PLR in the WT mouse. It could be argued that opioid inhibition of PLR is secondary to pupil constriction and thus decreased photic stimulation of the retina; however, this seems unlikely as in species where resting pupil constriction is seen secondary to opioids; e.g., in cats [11] and humans [9], opioids continue to inhibit the PLR over a wide range of pupil size. Moreover, while intense blue irradiance is still capable of driving the PLR in the face of DAMGO, this is not surprising given prior studies in which elimination of 97% of ipRGCs in the mouse resulted in incomplete PLR in response to low light intensity but did not prevent full pupil constriction in response to high light intensity [28]. In addition, the slowing of the blue light response of WT mice following intraocular DAMGO administration compared to control is in accordance with previous studies in humans in which opioids decreased the constriction velocity of the PLR [4]. The absence of DAMGO’s effect in McKO mice indicates that although MOR expression is not restricted to ipRGCs in the mouse retina [39], MORs expressed by ipRGCs are necessary and sufficient to mediate opioid action on the bright-blue-light-evoked PLR.

It is of note that the PLR in response to bright blue irradiance was greater in the MKO mice compared to the McKO mice. As previously mentioned, synaptic inputs onto ipRGCs downstream of rods and cones, including ON/OFF bipolar cells and amacrine cells, have been shown to extend the dynamic range of ipRGCs in both the intensity and temporal frequency domains [40]. Given that our prior work showed that MOR action in ipRGCs reduces excitability without affecting phototransduction [33], DAMGO is expected to reduce ipRGC signaling both when driven by rod/cone inputs and by the intrinsic melanopsin phototransduction pathway under bright light conditions. The greater pupillary constriction in the MKO group vs. the McKO group suggests that in the McKO group, in which MORs are absent from ipRGCs alone, opioids may be exerting inhibitory effects on elements of the retinal circuit downstream of the rod-cone photoreceptors [41], which are relevant for the integrated rod–cone and melanopsin-mediated PLR in response to bright blue irradiance [28], or alternatively, there may be centrally mediated endogenous opioid effects that are absent in MKO animals. The dark-adapted PLRs of MKO, McKO, and WT control mice were not significantly different; this may be due to compensatory mechanisms developed in the knockout mice from birth. CTAP’s enhancement of the rod/cone-driven PLR in the dark-adapted retina and melanopsin-driven PLR under photopic conditions in WT mice is consistent with our prior finding that the MOR-selective antagonist CTOP both recovered and increased the intrinsic light responses compared to control of rat ipRGCs recorded on multielectrode array (MEA) [33]. Thus, the intraocular application of CTAP appears to mimic the loss of in-circuit opioid effects achieved via knockout of MORs.

### 3.2. Role for Endogenous Opioid Regulation of the PLR

Is there a physiologic role for endogenous opioid regulation of the pupil? Regarding resting pupil size, systemically applied enkephalins in rats [40] and mice [42] produce pupil dilatation that is antagonized by naloxone. However, in mice, it seems unlikely that endogenous enkephalins have a significant role in the physiologic control of resting pupil size as neither pure naloxone blockade nor prolongation of endogenous enkephalin half-life altered pupil diameter [42]. Nonetheless, given that separate neural mechanisms control pupil size vs. the PLR and that the effect of MORs on each is species-specific, endogenous opioids may yet have a physiologic role in the modulation of PLR.

Enkephalins [43,44,45] and β-endorphin [32] have been detected in the avian and mammalian retina. For these endogenous opioids to regulate the PLR, there must also be receptors for opioids on cells within the retinal circuit relevant to the PLR. Retinal opiate binding sites have been demonstrated in several species, including chick, rabbit, goldfish, rat, mouse, cow, toad, and skate [33,41,46,47]. It has been shown that opioid receptor subtypes facilitate different, stereospecific opioid effects on pupil control [48]. While substrate specificity is not exclusive, of the endogenous opioids, enkaphalins bind preferentially to δ-opioid receptors and β-endorphin to µ-opioid receptors (MORs) [49], and the latter’s effects on the PLR are the subject of this study.

Here, we found that DAMGO did not significantly impair static PLR stimulated by bright blue light in the dark-adapted retina but did negatively regulate rod-cone mediated PLR. This is not surprising given that the photoisomerization of only a few hundred melanopsin molecules is all that is necessary to trigger a PLR [50], and near total ablation of the ipRGC population does not prevent the PLR evoked by bright illumination [28]. Furthermore, given that CTAP significantly enhanced rod–cone-mediated PLR in the dark-adapted retina but not melanopsin-mediated PLR in the light-adapted retina, the data suggest that endogenous opioids are more likely to present in the dark-adapted retina and exert inhibition on PLR, thereby allowing more low-irradiance light through the pupillary aperture to allow for improved vision during night hours. Together, our findings suggest that in the mouse retina, release of endogenous opioids, and specifically β -endorphin, might be regulated by light. This would be consistent with an intercellular feedback loop formed between the endogenous opioid system and dopamine release proposed initially for avian retina [51]: light triggers dopamine release that in turn tunes the retinal circuit for synaptic information processing under photopic conditions [52], and opioids that are released under low-light (scotopic) conditions serve as a dark switch by inhibiting dopamine release. The model receives support from studies showing reduction of retinal dopamine release by opioids in birds [53], turtle [54], and rabbit [55]. In addition, total opioid levels in murine brains are increased in the late afternoon [56], and pain-induced plasma β-endorphin levels peak at midnight [57]. In rat, there is also an increased degree of opiate receptor binding at night [58,59]. An increase in nighttime retinal opioid levels and binding to MORs on ipRGCs could account for the previously documented nighttime reduction in the ipRGC-driven post-illumination pupil response in humans [60,61,62,63], in addition to the results observed in the present study. A related point is that a relatively high level of dopamine has been detected in the vitreous in mammals, which, by acting on various types of dopamine receptors in the ciliary body, may exert a complex regulation of intraocular pressure (IOP) and, in turn, PLR [64].

Might opioids also affect the intrinsic PLR (iPLR), i.e., ipsilateral pupil constriction in response to photic stimulation of the retina without input to the brain? Recent studies showed putative M1 ipRGC processes to reach the ciliary muscles and that the iPLR is driven by melanopsin signaling from ipRGCs [60,61,62,63]. Together with our prior data showing MOR expression on ipRGC processes, [33], it is tempting to speculate that opioid action on the ciliary muscles might not be independent of ipRGCs. However, the effects of opioids on the iPLR are beyond the scope of the current study as we were only able to analyze the eye contralateral to the injected eye as reflex uveitis secondary to the injection procedure precluded the analysis of local opioid effects on the iPLR in the injected eye.

### 3.3. Considerations for Human Clinical Practice

How might opioids modulate the PLR in the clinical setting? Converging lines of evidence suggest that systemically applied opioids could act on MORs expressed by ipRGCs in the retina. For example, opioids, including morphine and methadone, cross the tight retina/blood barrier and accumulate in the vitreous humor of the eye [34,35] at concentrations high enough to trigger cellular effects via activation of MORs [65,66]. Morphine (0.30 µg/mL) and methadone (0.11 µg/mL) have been detected in the vitreous of opioid-dependent individuals [34,35]. Drugs administered via intravitreal injection are known to alter the activity of retinal neurons [67]; thus, intravitreal opioids are expected to activate opioid receptors in the retina, including those expressed by ipRGCs [33]

It is of note that opiate alkaloids lower IOP and in turn cause pupil constriction in the rabbit by acting on mu3 type opioid receptors via NO [58,68,69] and CO [70] generation. Along these lines, it is important to point out that delta [71] and kappa [72] opioid receptor activation have also been shown to reduce IOP and resting pupil diameter. However, DAMGO in our hands reduced the pupil constriction, so the reported effects cannot be explained by activating either delta or kappa receptors to reduce IOP. Furthermore, mu3-type opioid receptors are insensitive to peptide MOR agonist such as DAMGO, [73], so it is unlikely that in our experiments NO and/or CO-dependent mechanisms were triggered. Nonetheless, these opiate effects altering IOP should be considered in human patients receiving chronic morphine or methadone treatments.

Chromatic pupillometry is now utilized for the differentiation of retinal disease (inner vs. outer) and optic nerve disease in both human and veterinary medicine [74,75,76]. The PLR is utilized in non-ophthalmic applications as well, with melanopsin-mediated PLR deficits considered an indicator for increased vulnerability to major depressive disorder in low light conditions [77]. Given the prevalence of therapeutic opioid use, opioid modulation of the PLR should be taken into consideration when interpreting the results of diagnostic pupillometry. On the other hand, akin to resting pupillary diameter serving as an indicator of systemic opioid administration, altered PLR dynamics may represent a novel biomarker for response to/efficacy of opioid (ab)use therapy. For example, pupillary unrest under ambient light (PUAL) is depressed by opioids, and there is a positive correlation between higher levels of post opioid administration PUAL changes and greater analgesia [78].

## 4. Materials and Methods

### 4.1. Animals

All animals used in these studies were handled in compliance with the Institutional Animal Care and Use Committees of Colorado State University (Protocol 18-8395A, 28 January 2019) and in accordance with the ARVO Statement for the Use of Animals in Ophthalmic and Vision Research. Animals were housed under a 12:12 light/dark (LD) cycle. Food and water were made available ad libitum. Three strains of mice were used. C57BL/6J (stock# 000664, Jackson Labs, Bar Harbor, ME, USA) mice, in which opioid-addiction-relevant behaviors are robust [79], were used as wild-type (WT) controls. Mice lacking functional MORs globally (B6.129S2-Oprm1tm1Kff/J, MKO for short) were purchased from Jackson Labs (stock# 007559). We generated a cell-specific, conditional MOR KO mouse line, specifically, in which only ipRGCs were lacking MORs (McKO for short) as described before [80], by crossing a well-characterized mouse line expressing Cre recombinase upstream of the melanopsin coding sequence (*Opn4*) (Tg(Opn4-cre)SA9Gsat/Mmucd or *Opn4::Cre* for short, sock # 036544-UCD, MMRRC) with mice where exon 2 and 3 of the MOR gene (*Oprm1*) are flanked by a loxP site (“floxed μ” or *Oprm1*^fl/fl^). *Oprm1*^fl/fl^ breeders were generously provided by Dr. Brigitte Kiefer (Douglas Research Center, McGill University). McKO mice had *Cre* on one, and “floxed μ” on both alleles. RNA in situ hybridization to verify MOR knock-down in ipRGCs (McKO) was performed using RNAscope technology (Advanced Cell Diagnostics, Newark, CA, USA) as previously described [81].

### 4.2. In Vivo Pupillometry

Adult male and female mice were dark-adapted for 15 min. PLR was tested on mice that were either awake or maintained on a very light plane of anesthesia with isoflurane [16]. There was limited bias due to handling stress or anesthetic plane as reproducible control pupil sizes were obtained prior to each stimulus. Green (525 nm) and blue (470 nm) light stimuli were generated by LEDs (American Bright Optoelectronics, Chino, CA, USA and Digi-Key, Thief River Falls, MN, USA, respectively) and were projected to the right eye by means of a liquid light guide. The LED voltage and ON-OFF timing were controlled by a Master 8 programmable pulse generator (AMPI, Jerusalem, Israel). The light intensity at the level of the cornea was calibrated before the experiments with an optical meter (model 1918-C, sensor 918D-SL-OD3; Newport, Irvine, CA, USA). Intermittent light enhances pupillary constriction responses and prevents adaptation [82]; accordingly, we delivered the 1 min long light stimulation at 2 Hz to the right eye, while recording PLR in the left eye at 30 frames/sec with an infrared camera. Control stationary pupil measurements were taken 1–10 s before the stimulation was begun. Stationary PLR was recorded after 1 min of intermittent light stimulation. Stationary recovery values of the pupil size were recorded ~2 min after the termination of light stimulation protocol. Pupil area was measured off-line at 1 s intervals using an open source image analysis software (NIH ImageJ, https://imagej.nih.gov/ij/index.html). Similar to prior studies [29], to correct for individual variation in dark-adapted pupil area, pupil sizes during illumination were calculated as the percentage of the average of the stationary control and recovery pupil sizes.

It is of note that recent work with dynamic pupillometry comparing WT vs. rodless or coneless mice has demonstrated that rods contribute to blue-light PLR and low- and medium-intensity red-light PLRs, while cones drive the initial rapid dilation of low-intensity blue light PLR [16]. However, the focused goal of this study was to clearly delineate MOR mediation of classical photoreceptor vs. ipRGC input on the (stationary) PLR without subdividing the classical photoreceptor inputs into those of rods vs. cones. As previously discussed, it is difficult to chromatically make a distinction between the relative contributions of rod and cone input to ipRGCs [17,25,26,27,28], given that the wavelength sensitivity of rods and green cones in mice closely overlap at λ_max_ 498 nm and 508 nm, respectively [82]. For this reason, most landmark studies assessing the relative contribution of classical photoreceptor and melanopsin photoresponses to ipRGC physiology pool rod and cone inputs together as a collective outer retinal input, utilizing high- vs. low-intensity light stimulus protocols [82]. Additional laboratories have utilized red light (630 nm, luminance 200 kcd/m^2^) to elicit PLRs in mice; however, without the benefit of genetic knock-out mice, the resultant PLR was still considered to be a combined, rod–cone-mediated PLR [58,67,68]. Given the above, for rod/cone stimulation, we elected to use green light (10^10^ photons/cm^2^/s at 525 nm), expected to saturate rod and activate green cone opsin [36], which is considered to be mesopic intensity [83], and 10^14^ photons/cm^2^/s at 470 nm to activate ipRGCs directly [17,18].

MOR-selective agonist [D-Ala^2^, MePhe^4^, Gly-ol^5^]-enkephalin (DAMGO) or the MOR-selective antagonist D-Phe-Cys-Tyr-D-Trp-Arg-Thr-Pen-Thr-NH_2_ (CTAP) (Tocris, Minneapolis, MN, USA) (2 mg/mL each) were administered via unilateral intravitreal injection (2 µL/eye) under isoflurane anesthesia following application of topical 0.5% proparacaine [84]. Controls received saline (2 µL/eye). Mice were dark-adapted for 15 min. PLR was tested on mice maintained on a light plane of anesthesia with isoflurane, in the same fashion as for the control series, with the light stimulus being delivered to the sham/opioid-treated right eye and PLR recorded from the contralateral left eye. Each animal was tested only once with a given light stimulus; thus, “*n*” stands for the number of mice tested throughout the paper.

Our previous multielectrode array data shows that the maximal effect of DAMGO for reducing ipRGC response was reached at ~1 µM. [33]. Intravitreal injection of 2 µL of 2 mg/mL DAMGO will result in ~100 µM DAMGO concentration in the vitreous considering equal distribution in the estimated total vitreous volume (~20 µL) of the mouse eye [85]. Thus, even if some drug reflux took place during and following the injection [86], the intravitreal concentration of DAMGO is expected to produce maximal inhibition of light-evoked ipRGC spiking and, in turn, inhibition of PLR. Existing evidence supports this: pharmacological inhibition of melanopsin with opsinamides inhibited ipRGC firing by about 50% and reduced bright-light-triggered PLR in rodless/coneless mice by about 50%, without affecting PLR evoked by dim intensities (i.e., rod-cone mediated PLR) in WT mice [26]. The unilaterally delivered intravitreal DAMGO is expected to remain below the dose necessary to cause cellular effect by acting on MORs anywhere else in the body of the mouse, [66], considering the volume of blood (~1.5 mL/mouse) and the extracellular spaces outside of the blood vessels (i.e., brain, or the vitreous of the contralateral eye) assuming even distribution of DAMGO across the mouse within the duration of the experiments (10–15 min).

### 4.3. Statistical Analysis

All data were analyzed using SigmaPlot11 (version 11; Systat Software, San Jose, CA, USA). Specific statistical comparisons are described in-text. Data are presented as mean ± SEM, and *p* < 0.05 considered significant.

## 5. Conclusions

Our results indicate that intraocular opioids acting on MORs of ipRGCs are negative modulators of the PLR and are suggestive of a potential increase in endogenous opioid concentrations in the dark-adapted retina. Future studies should investigate the effect of systemic opioid administration on both the static and dynamic PLR, as this may have a significant impact on the interpretation of diagnostic pupillometry and serve as a potential biomarker of systemic opioid effect.

## Data Availability

The data (video files of pupil constrictions) analyzed and presented in this study are available on request from the corresponding author.

## Abbreviations

MOR	µ opioid receptor
ipRGC	intrinsically photosensitive retinal ganglion cell
PLR	pupillary light reflex
DAMGO	[D-Ala^2^, MePhe^4^, Gly-ol^5^]-enkephalin
CTAP	D-Phe-Cys-Tyr-D-Trp-Arg-Thr-Pen-Thr-NH_2_
OPN	olivary pretectal nucleus
OMN	oculomotor nucleus
EWN	Edinger-Westphal nucleus
WT	Wild-type mouse
MKO	Mice lacking MORs throughout their body
McKO	Mice lacking MORs in ipRGCs
IOP	Intraocular pressure
PUAL	pupillary unrest under ambient light
